# Predicting Engagement While Playing Computer Games in Older Adults With and Without Dementia

**DOI:** 10.1093/geroni/igaa057.3180

**Published:** 2020-12-16

**Authors:** Antonio Miguel-Cruz, Daniel Alejandro Quiroga-Torrez, Adriana Maria Rios-Rincon, Christine Daum, Ruby De Jesus, Lili Liu, Eleni Stroulia

**Affiliations:** 1 University of Alberta, Edmonton, Alberta, Canada; 2 Universidad del Rosario, Bogotá, Distrito Capital de Bogota, Colombia; 3 University of Waterloo, Waterloo, Ontario, Canada

## Abstract

The purpose of this study was to determine what factors predict the level of engagement of older adults, with and without dementia, while playing computer games. Fourteen older adults with and without dementia (60%/40%) played a computer game over 16 sessions, each for 30 minutes. Variables included participants’ demographics, game-play data and environmental factors. Mixed fixed model for longitudinal data analysis design was used to determine how these variables predicted engagement. Five variables predicted engagement at a statistically significant level: Participant’s performance (B1=+0.16, p<0.03), age (B2=+0.20, p<0.00), previous experience with computer games (B3=+1.021, p<0.02), positive emotions (B4=+0.16, p<0.00), and distractions (noise) during gameplay (B5=-1.07, p<0.05). Cognitive impairment and general health status were correlated with engagement, but these correlations were not statistically significant. Previous experience using computer games and distractions during gameplay were the most important predictors of engagement while older adults with and without dementia played computer games.

